# Small intestinal microbial fiber metabolism dysfunction in celiac disease

**DOI:** 10.21203/rs.3.rs-6572358/v1

**Published:** 2025-05-12

**Authors:** M Wulczynski, M Constante, HJ Galipeau, J-J Blom, GH Rueda, N El-Chaar, DK Superdock, S Jiang, LA David, JA Murray, MG Surette, D Armstrong, MI Pinto Sanchez, P Bercik, A Caminero, EF Verdu

**Affiliations:** 1- Farncombe Family Digestive Health Research Institute - McMaster University, Hamilton, ON, Canada; 2- Department of Molecular Genetics and Microbiology, Duke University School of Medicine, Durham, NC, USA.; 3- Division of Gastroenterology and Hepatology, Mayo Clinic, Rochester, MN, USA.

## Abstract

Celiac disease (CeD) is an immune-mediated condition driven by dietary gluten resulting in small intestinal mucosal inflammation and injury, along with myriads of symptoms. The only treatment is a lifelong gluten-free diet (GFD) and although most patients improve, the restriction can lead to nutrient deficiencies including fiber. While duodenal microbiota is altered in CeD, its fiber metabolic capacity is unknown. Here we show that both active and treated CeD patients had impaired microbial fiber metabolism in the small intestine which associates with depletion of the carbolytic taxa *Prevotella spp*. Colonization of germ-free mice with *Prevotella spp* increased small intestinal short chain fatty acids (SCFA). In gluten-sensitized mice expressing the celiac risk gene, HLA-DQ8, an inulin-supplemented diet facilitated microbial carbolytic function, SCFAs production and accelerated mucosal healing in the small intestine during GFD. The results support clinical investigations of dietary fiber supplementation and microbial carbolytic function to enhance responses to GFD in CeD.

## Introduction:

Celiac disease (CeD) is an immune-mediated disorder driven by related prolamin proteins termed “gluten” in wheat, rye, and barley^1^. The disease affects the proximal small intestine, leading to intraepithelial lymphocyte infiltration, villus blunting, and crypt hyperplasia. CeD has autoimmune traits, including HLA-DQ2 or -DQ8 association, anti-tissue transglutaminase 2 (TG2) autoantibodies, and immune-mediated intestinal epithelial cell destruction^1,2^. While about 30% of the global population is genetically predisposed, only 2–3% of those at risk develop the condition^3,4^. This, tied to the rapid growth in prevalence and incidence in the past decades, suggests that there are environmental determinants in addition to gluten^5,6^.

Longitudinal studies in genetically at-risk populations suggest that gastrointestinal infections at early life, especially when combined with high gluten intake, are associated with higher risk to develop CeD^6–8^. Cross sectional studies have demonstrated differences in microbiota composition and function in the small intestine of active CeD^9–11^. Indeed, diet is a major driver of microbiota establishment and function, and recent studies have suggested that higher consumption of fiber-rich foods such as vegetables is associated with lower odds of developing celiac autoimmunity^5,12^. A recent reanalysis of the large longitudinal cohort study, *The Environmental Determinants of Diabetes in the Young* (TEDDY), suggests an association between increased fiber intake in early life (<3 years) and reduced risk of CeD^13^. In the colon, short-chain fatty acids (SCFAs; such as acetate, propionate, and butyrate), produced by microbial fiber metabolism, have been linked to gastrointestinal health through improved motility, anti-inflammatory activity, and microbiome modulation, primarily mediated through G-protein coupled receptors, GPR41 and GPR43, which are widely expressed on immune and epithelial cells^14,15^. However, the role of microbial fiber metabolism and SCFA production in the small intestine in the context of CeD, remains understudied.

The only current treatment for CeD is the lifelong elimination of gluten. While a gluten-free diet (GFD) is effective in most patients, mucosal healing is slow and often impeded by rapid reactivation of memory cells, likely related to accidental gluten exposure. Although the GFD is usually low in fiber^16^, especially when composed of processed gluten-free products, little is known about the fiber metabolic capacity of CeD microbiota along the gastrointestinal tract. Indeed, increased fiber intake is often recommended to CeD patients on GFD to manage constipation, without mechanistic insight on microbial carbolytic capacity or its impact on inflammation.

Our study reveals that patients with active and treated CeD had impaired microbial fiber metabolism in the small intestine independently of differences in fiber intake. Carbolytic taxa depleted in CeD include *Prevotella spp*, known fiber degraders^17^. Colonization of germ-free mice with *Prevotella spp* increased SCFA in the small intestine. In gluten-sensitized NOD/DQ8 mice, the soluble fiber inulin accelerated mucosal healing by supporting carbolytic activity of the small intestinal microbiota. Our results support intervention with dietary fibers, such as inulin, in CeD patients alongside the GFD, to improve mucosal healing so long as patients harbor the necessary microbial function.

## Results

### Patient demographics.

A total of 16 CeD patients diagnosed within the previous 3 months (newly diagnosed CeD), 11 CeD patients on a >2 years GFD (T-CeD) and 26 healthy controls were recruited for the study. Details on participant demographics, clinical characteristics and samples collected are summarized in Table 1.

### Microbial carbolytic function in the small intestine was lower in CeD.

**Figure 1:**
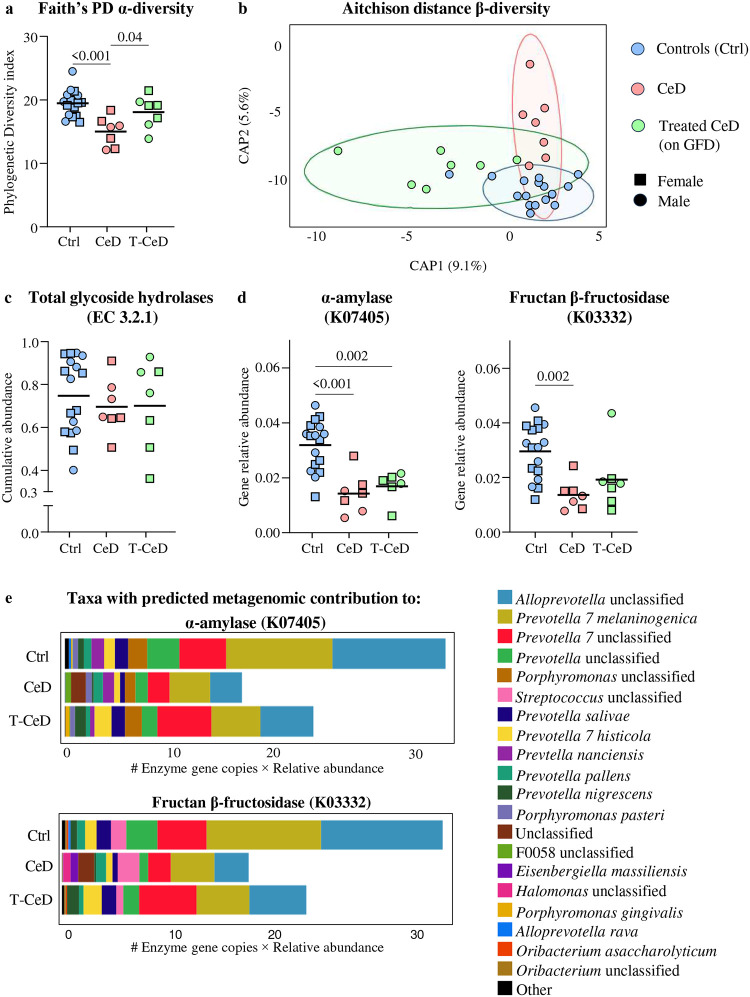
Predicted decreases in duodenal microbial carbolytic enzymes in celiac disease versus healthy controls. **a** Faith’s Phylogenetic Diversity α-diversity of duodenal aspirate microbiota. **b** Aitchinson distance β-diversity plot of microbial profiles from duodenal aspirates from controls. Samples were distinctly separated by group using PERMANOVA (*p*<0.01). **c** Cumulative abundance of microbial glycoside hydrolase genes in the total PICRUSt2 predicted metagenome from duodenal aspirates. **d** Relative abundance of α-amylase (K07405) and fructan β-fructosidase (K07405) gene copies from duodenal aspirates. **e** PICRUSt2 predicted metagenomic contribution of top bacterial genera to genes of interest. Data for **a, c, d** were compared using one-way ANOVA followed by Tukey’s test. For all data, healthy controls (Ctrl; blue, n= 17), celiac disease (CeD; red, n= 7), and treated celiac disease (T-CeD;green, n= 7), and sex was represented by females (squares) and males (circles).

Microbiota composition of duodenal aspirates was assessed using 16S RNA sequencing. Duodenal α-diversity was lower in CeD than in controls or T-CeD (Fig. 1a), and β-diversity confirmed distinct profiles between the three groups (Fig. 1b). PICRUSt2 analysis revealed no differences in total glycosidase hydrolase genes (EC 3.2.1) between CeD, T-CeD and controls (Fig 1c), however, microbial genes encoding α-amylase (EC 3.2.1.1; K07405) and fructan β-fructosidase (EC 3.2.1.80; K03332), a fiber degrading enzyme^18^, were lower in CeD and T-CeD versus controls (Fig. 1d). The metagenomic contributions to these genes from top genera are visualized in Fig. 1e. The top 4 predicted contributors belonged to *Prevotellaceae* family which were less abundant in CeD and T-CeD versus controls (Supplementary Fig 1). Overall, microbial alterations associated with CeD were characterized by lower predicted microbial carbolytic function, independently of treatment with GFD.

### Diet does not fully explain lower microbial carbolytic function in CeD.

**Figure 2:**
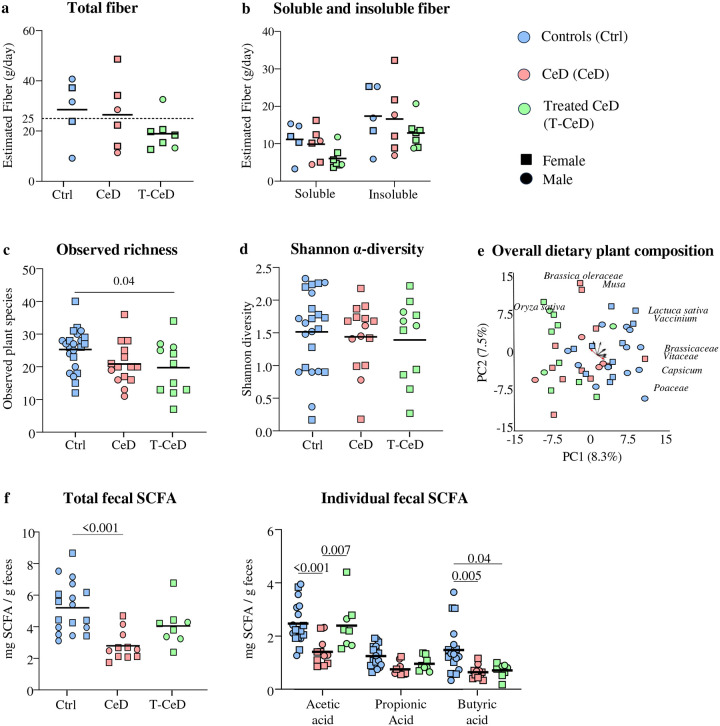
Patients with CeD have lower fecal SCFA than T-CeD and healthy controls. **a** Estimated total daily fiber consumption in controls (blue, n= 5), active CeD (red, n= 6), and T-CeD (green, n= 7). Dashed line represents the daily recommendation from Health Canada. **b** Calculated soluble and insoluble fractions of estimated total fiber in healthy controls (blue, n= 5), active CeD (red, n= 6), and T-CeD (green, n= 7). **c** Number of distinct plant taxa in healthy controls (blue, n= 23), CeD (red, n= 15), and T-CeD (green, n= 11); Control vs T-CeD: β = −5.98±2.78, p < 0.05. **d** Shannon α-diversity of plant taxa in healthy controls (blue, n= 23), CeD (red, n= 15), and T-CeD (green, n= 11). **e** Principal component analysis of dietary plant composition in healthy controls (blue, n= 23), CeD (red, n= 15), and T-CeD (green, n= 11). Vectors identify top 9 taxa driving variation with red emphasis on *Oryza sativa* and *Poaceae*. Overall dietary plant composition was influenced by patient group (PERMANOVA; df = 2, F = 2.04, R^2^ = 0.080, p = 0.001), and specifically between control vs T-CeD: PERMANOVA; df = 1, F = 3.94, R^2^ = 0.110, adjusted p < 0.05. **f** Fecal SCFA in healthy controls (blue, n= 18), CeD (red, n= 11), and T-CeD (green, n= 8). For all data multiple groups were compared using one-way ANOVA followed by Tukey’s test, and sex was represented by females (squares) and males (circles).

Diet was investigated to determine whether altered microbial predicted function was associated with fiber consumption. In the majority of participants, the fiber intake was below the minimum recommendation for fiber set by Health Canada (25 g/day), based on food frequency questionnaire (FFQ^19^), with up to 86% of T-CeD being fiber-deficient (Fig. 2a). Although a similar trend was observed for total fiber, there were no statistical differences between the calculated soluble and insoluble fiber fractions between groups (Fig 2b). To compare dietary patterns at time of sample collection, fecal plant DNA was analyzed using FoodSeq^20^, which showed lower observed plant richness (total plant species) in T-CeD group compared with controls (Fig 2c), although Shannon diversity was similar (Fig 2d). The post-hoc analysis of overall dietary plant composition revealed a significant difference between T-CeD and controls (Fig. 2e), characterized by patterns such as opposing loadings of *Oryza sativa* (rice) and *Poaceae* (rye, wheat family), consistent with the elimination of gluten-containing foods. Overall, food sequencing patterns demonstrated GFDs were highly individualized (Supplementary Fig. 2). Patient reported gastrointestinal symptom scores (GSRS; total score and individual sub-scores) did not correlate with fiber intake, suggesting no obvious relationship with symptoms. Thus, while T-CeD participants had a shift in plant DNA signatures, overall fiber intake was not significantly different versus CeD or controls.

To assess microbial metabolism of fiber in our cohorts, fecal SCFA were measured as a marker of fiber fermentation using gas chromatography mass spectrometry (GC/MS). CeD patients had lower total SCFA compared with controls (Fig. 2f). Specifically, acetic acid levels were lower in CeD than in controls or T-CeD, and butyric acid levels were lower in CeD and T-CeD groups as compared to controls. While T-CeD had the lowest fiber intake of the 3 groups, these patients had modestly higher SCFA levels than CeD. The results suggest T-CeD partially recovers microbial carbolytic capacity, despite the sub-optimal amount of fiber ingested by these patients, which may be related to histological amelioration.

### Inulin accelerates recovery of gluten-immunopathology in NOD/DQ8 mice on GFD.

Mice with transgenic expression HLA-DQ8 gene in non-obese diabetic background (NOD/DQ8) were sensitized with gluten as previously described^21^ to determine timing of mucosal recovery from gluten-immunopathology after 2, 6, 10, and 24 weeks of GFD (Supplementary Fig. 3a). CD3^+^ IELs, villus to crypt (V/C) ratios and composite VCIEL score normalized by week 10 of GFD in previously sensitized mice (Supplementary Fig. 3). However, small intestinal antibodies against gliadin or TG2 remained elevated in some mice (Supplementary Fig. 4).

**Figure 3:**
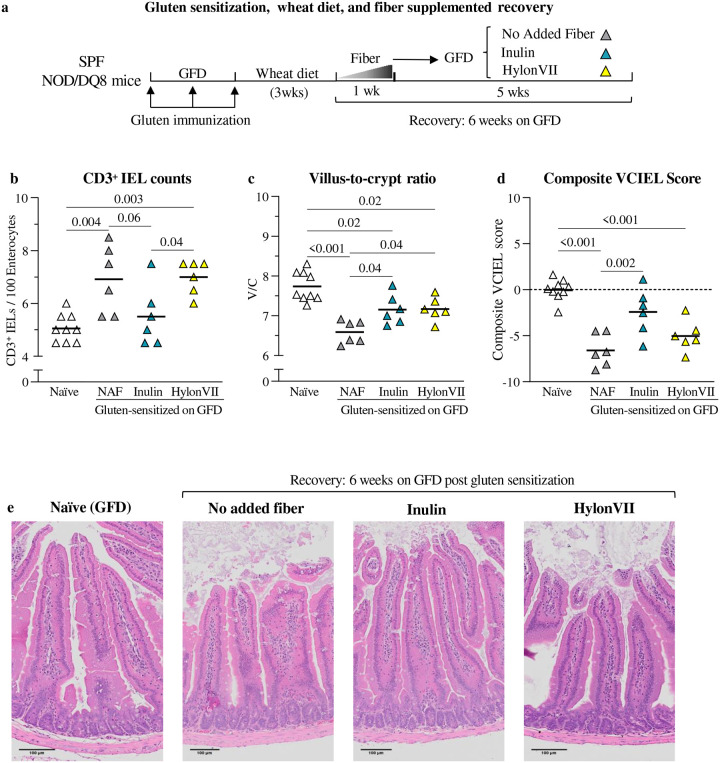
Inulin accelerates histological recovery in gluten-sensitized SPF NOD/DQ8 mice **a** Schematic protocol for gluten sensitization, wheat diet, and different fibers supplemented during recovery on GFD. Naïve (non-sensitized controls, no added fiber GFD, white n= 9), no added fiber (grey n= 6), inulin (blue n = 6) and HylonVII (yellow n= 6). **b** CD3^+^ intraepithelial lymphocytes numbers after six weeks of recovery by diet (n= 6 / experimental group). **c** Villus-to-crypt ratio after six weeks of recovery by diet (n= 6 / experimental group). **d**, Composite VCIEL scores after six weeks of recovery by diet (n= 6 / experimental group). Dashed line represents the baseline determined from naïve controls. **e** Representative histological images of jejunal tissue after six weeks of recovery by diet. For all data, multiple groups were compared using one-way ANOVA followed by Tukey’s test.

Once determined that sensitized NOD/DQ8 mice normalized histological parameters at week10 of GFD, the effect of fiber supplementation was investigated. Additional sensitized NOD/DQ8 mice (Fig 3a) were given GFD with no added fiber or supplemented with either inulin (52g/kg) or HylonVII (136g/kg) supplemented diets for 6 weeks (described in Table 2). Compared with non-sensitized controls (naïve), sensitized mice had higher CD3^+^ IELs (Fig 3b), and lower V/C ratio (Fig 3c) and composite VCIEL score (Fig 3d). Supplementation with inulin during GFD improved all histological parameters versus the no added fiber group. HylonVII did not normalize IELs, but mice had higher V/C ratios versus no added fiber group. Neither of the fiber diets normalized antibodies in intestinal content at 6 weeks follow-up. However, all mice became seronegative after 12 weeks of inulin or HylonVII (Supplementary Fig. 4).

### Inulin increases small intestinal SCFA without major changes in microbial profiles.

**Figure 4:**
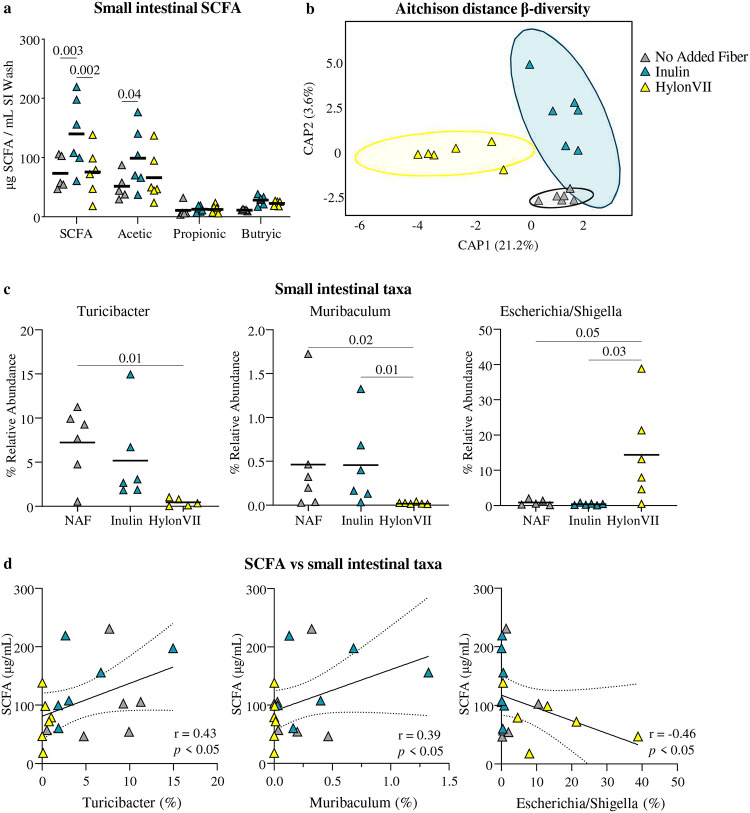
Inulin increases small intestinal SCFA in gluten-sensitized SPF NOD/DQ8 mice **a** Small intestinal SCFA after 6 weeks of recovery with no added fiber GFD, inulin (IN), or HylonVII (RS). **b** Aitchinson distance β-diversity plot of microbial profiles from mice after six weeks of recovery on no added fiber GFD (grey), inulin (blue) and HylonVII (yellow). Samples from HylonVII were distinct from GFD and inulin groups using PERMANOVA (*p*<0.01). **c**, Relative abundances of *Turicibacter*, *Muribaculum*, and *Escherichia/Shigella* in the duodenum after 6 weeks of recovery. **d** Small intestinal SCFA levels positively correlated with abundances *Turicibacter* and *Muribaculum*, and negatively correlate with Escherichia/Shigella by Pearson’s correlation. For **a, c,** multiple groups were compared using one-way ANOVA followed by Tukey’s test. For all data n= 6 / group.

Inulin supplementation during GFD, but not HylonVII, was associated with higher SCFA, primarily acetic acid, in the small intestine of SPF NOD/DQ8 mice, compared with no added fiber (Fig. 4a). There were no differences in profiles between inulin and no added fiber groups (Fig. 4b). HylonVII supplemented mice clustered separately with lower relative abundance of *Turicibacter* and *Muribaculum*, and higher *Escherichia/Shigella* (Fig. 4c). SCFA correlated positively with *Turicibacter* and *Muribaculum* and negatively with *Escherichia/Shigella* relative abundances (Fig. 4d). Additional details on microbiota composition are provided in Supplementary Fig. 5.

### Inulin and Prevotella boost SCFA production in the small intestine.

**Figure 5:**
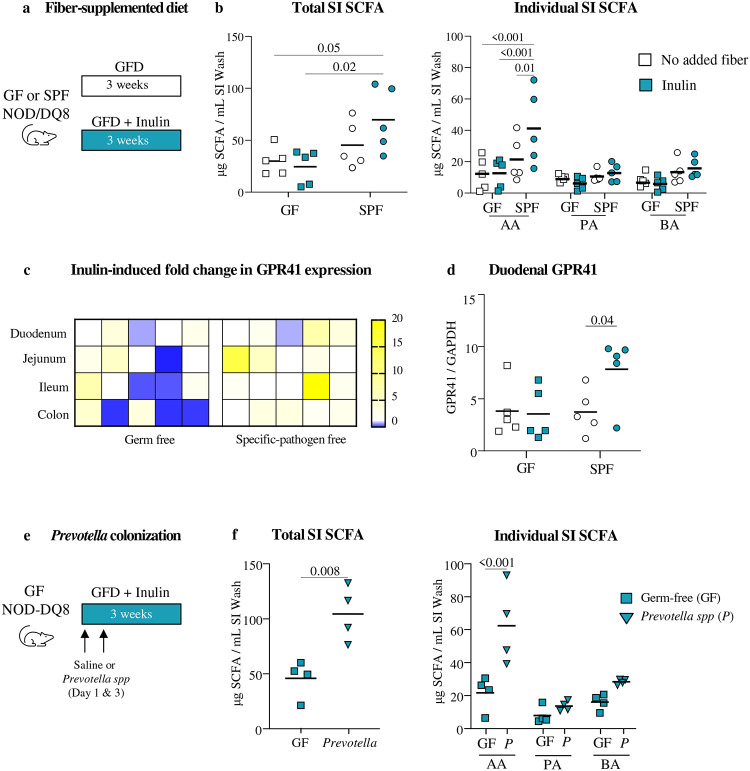
SPF and gnotobiotic mice colonized with *Prevotella spp* produce SCFA from inulin *in vivo*. **a** Schematic protocol for diet intervention non-sensitized germ-free (GF, squares) and specific-pathogen free (SPF, circles) NOD/DQ8 mice were fed either GFD with no added fiber (grey) or supplemented with inulin (blue) for 3 weeks (n= 5 / group). **b** Small intestinal SCFA after 3 weeks of no added fiber GFD (white) or inulin diet (blue) in GF (squares) and SPF (circles) mice. Multiple groups were compared using one-way ANOVA followed by Tukey’s test. **c** Relative change in SCFA receptor *Gpr*41 measured in tissue samples from the duodenum, jejunum, ileum, and colon of GF and SPF mice when supplemented inulin. Data is represented as the fold-change in *Gpr*41 expression when fed inulin compared to no added fiber diets as control for GF and SPF mice. **d** Expression of *Gpr*41 in the jejunum. For each location, a Student’s T Test compared *Gpr*41 expression in mice fed GFD with no added fiber or with inulin; for GF and/or SPF mice. **e** Schematic protocol for *Prevotella* colonization of GF NOD/DQ8 mice (n= 4 / group). Mice were given inulin diet and colonized by oral gavage with either *Prevotella* cocktail (triangles) or saline (squares; vehicle control) on days 1 and 3. **f** Small intestinal SCFA of GF (square) and *Prevotella spp*. colonized mice after three weeks of diet. Data was compared using Student’s T test. AA - Acetic acid; PA - Propionic acid; BA - Butyric acid.

To further investigate small intestinal microbial fiber metabolism *in vivo* we first compared SCFA production in germ-free (GF) versus SPF NOD/DQ8 mice in response to inulin (Fig 5a). Inulin increased total small intestinal SCFA in SPF versus GF mice (Fig. 5b), confirming that fiber metabolism required microbiota presence. This was driven by increases in acetic acid, with no differences in propionic or butyric acids. As expected, the magnitude of SCFA increase was 10-times higher in feces than the small intestine, also evidencing differences in the main SCFA produced between the two sites (Supplementary Fig. 6a). Next, to investigate host responses to local SCFA availability, expression of SCFA receptors, GPR41 and GPR43, was assessed along the intestinal tract. Inulin associated with higher *Gpr*41 and *Gpr*43 expression at most small intestinal sites, including the duodenum (Fig. 5c, d). No differences in SCFA receptors were observed in GF mice after inulin supplemented diet.

Finally, based on our observation that fiber-degrading bacteria from the *Prevotellaceae* family were depleted in the duodenum of CeD patients, GF NOD/DQ8 mice were colonized with a cocktail of 10 *Prevotellaceae* members (Fig. 5e, Table 3). *Prevotella*-colonized mice, but not GF mice, supplemented with inulin had higher small intestinal SCFA (Fig 5f). The results demonstrate that small intestinal taxa, such as *Prevotellaceae*, can produce SCFA from dietary fiber in the small intestine when the appropriate substrate in present.

## Discussion:

Management of chronic gastrointestinal conditions through dietary approaches is common and is related to the ability of some food substrates to modulate gut microbiota composition or function in a beneficial^15^ or detrimental manner^22^. Fiber and its metabolites produced in the lower gastrointestinal tract as a result of microbial metabolism, are frequently associated with improved gastrointestinal health^14,15^, although current dietary guidelines do not always provide evidence-based recommendations for specific diseases. In CeD, inflammation occurs in low bio-mass sites, primarily in the duodenum^23^. Thus, the impact of fiber metabolism on gluten-driven inflammation has not been investigated, despite a high fiber intake often being recommended to newly diagnosed patients alongside the only current therapy, a strict GFD. Here we report that key fiber-degrading bacteria are depleted in the small intestine of patients with CeD both before and after treatment with a GFD, impairing microbial carbolytic activity. Furthermore, in genetically predisposed mice sensitized with gluten, we identify a fiber-specific effect that increases small intestinal SCFA production and accelerates recovery from gluten immunopathology after GFD.

Given that GFD are often low in fibre, especially when relying on processed gluten-free products, CeD patients are advised to increase fiber intake through diet or supplements, to avoid symptomatic complications such as constipation^16,24^. Furthermore, results from different large-scale population based prospective studies have identified a potential role for fiber in CeD management beyond constipation. The *Norwegian Mother, Father, and Child Cohort Study* (MoBa) showed that higher maternal fiber consumption associated with lower risk of CeD in offspring^25^, which parallels the findings from the TEDDY cohort, where increased fiber during early life reduced risk as well^13^. While these studies identified interesting epidemiological associations, they did not investigate underlying mechanisms. Using a cohort of healthy controls, newly diagnosed CeD patients and T-CeD >2 years on GFD, we here show that many individuals have lower than recommended fiber intake which tends to further decrease when they adopt a GFD. This fiber deficit is consistent with the *Canadian Community Health Survey* that reported inadequate median fiber intake in over 35,000 healthy Canadians^26^. To further investigate differences in food intake between cohorts objectively, fecal plant DNA was assessed. Specific taxa such as *Poaceae* (wheat, rye family) and *Oryza sativa* (rice) were identified as opposing pattern drivers between controls and T-CeD. CeD patients had lower total SCFA than healthy controls, despite similar abundance and diversity of plant DNA in feces. T-CeD, had the highest estimated fiber deficiency, and demonstrated only partial increase in SCFA, which aligns with previous studies showing lower SCFA in untreated CeD children versus patients with partial restoration after the GFD.^27,28^ The reason for this is unclear, but could relate to some recovery of microbial function after improvement of small intestinal inflammation in T-CeD, which supports dietary intervention studies in this population.

Microbial changes in CeD have been described as factors that can contribute to disease initiation, perpetuation, and resolution^5^. Our results are consistent with previous descriptions that duodenal microbiota profiles differ between active CeD, T-CeD and healthy subjects. Although diagnostic and universal signatures have not been identified due to individual and methodological variability, some taxa are more commonly observed across different studies^9^. Here we found that members of *Prevotellaceae* family were less abundant in CeD and T-CeD versus controls with lower predicted microbial carbolytic function, independently of treatment with GFD. *Prevotellaceae*, a fiber degrading taxa^29^, has been previously reported to be lower in duodenal biopsies of both children and adults with CeD compared with controls^11^. *Prevotella spp* have also been involved in immunomodulation within the gastrointestinal tract and some species have been shown to ameliorate systemic autoimmunity in experimental autoimmune encephalomyelitis, models of collagen-induced arthritis and diabetes, with positive effects in phase one trials for autoimmune skin disease^30,31^.

To further investigate this mechanistically, we used NOD/DQ8 mice which express moderate immunopathology after sensitization with gluten and a bacterial adjuvant^21,34^. In this model, gluten-immunopathology can be exacerbated or improved, depending on the microbial community mice are colonized with^35,36^. We showed that an inulin-supplemented diet increased small intestinal SCFA in SPF NOD/DQ8 mice, specifically acetate, without altering microbiota composition, and that this was associated with faster histological resolution, to a greater extent than HylonVII. It is possible that inulin undergoes more rapid partial digestion in the small intestine compared with HylonVII, a type-2 resistant starch made of linear α−1,4 and branching α−1,6 linkages. While *Turicibacter* and *Muribaculum* were positively associated with small intestinal SCFA in inulin supplemented mice^37^, a higher relative abundance of the potentially proinflammatory taxa *Escherichia/Shigella*^38,39^ was observed in HylonVII fed mice. The latter could explain the lower degree of histopathology improvement versus inulin. Finally, colonization of germ-free mice with *Prevotella spp* increased small intestinal short chain fatty acids (SCFA) upon inulin supplementation. A previous seminal study postulated that long-term fiber inadequacy could cause loss of fiber-degrading taxa in the gut, which might not be restored by increased fiber without supplemental microbiota^32^. Thus, while some gluten-free products are now formulated with a higher fiber content^33^, overall our results indicate the potential need to optimize both substrate and microbial metabolizers during GFD, likely in a personalized manner.

Our study has some limitations. The FFQ was not specifically designed to assess GFD, and not all participants completed the questionnaire. However, we supplemented the FFQ with an objective dietary sequencing method, FoodSeq, which investigated plant DNA in fecal samples at the time of sample collection. We did not perform direct measurement of small intestinal SCFA from human aspirates because it is technically challenging. Instead, we predicted microbial small intestinal fiber metabolism in the human aspirates using PICRUSt2 and performed mechanistic studies in mice, including with humanized microbiota.

In summary, the duodenum constitutes a unique microbial niche which should be considered distinct from the rest of the intestine when investigating the role of fiber metabolism in the prevention and treatment of diseases that affect primarily the small bowel. Our study provides clinical evidence that the small intestinal microbiota in CeD contains less fiber-degrading bacteria, such as *Prevotellaceae* independently of diet and preclinical evidence that certain fibers could be used as adjuvant therapy for the GFD to promote mucosal healing, when metabolizing taxa are present.

## Methods:

### Human participants

Patients attending the McMaster University Celiac Clinic were recruited after signing informed consent. New cases of CeD (consuming gluten) were diagnosed based on positive serological markers, the presence of either HLA-DQ2 or HLA-DQ8, and evaluation of duodenal biopsies (n= 16). Treated CeD (T-CeD) included patients with a verified diagnosis, who were on GFD > 2 years and attending the clinic as part of their follow-up (n= 11). Healthy controls included patients attending the McMaster University Digestive Diseases Clinic with a history of gastroesophageal reflux disease without other symptoms, and/or having confirmed normal endoscopy and biopsy, negative celiac serology, and negative *Helicobacter pylori*. They also did not fulfill criteria for disorders of gut-brain interaction (DGBI) based on Rome IV criteria^40^. All participants were asked to complete a validated food frequency questionnaire (Victoria Cancer Council Dietary Questionnaire for Epidemiological Studies v2)^19^ to assess their habitual diet, and a validated gastrointestinal symptoms ratings score (GSRS)^41^ to assess symptoms. No participant had prescribed proton pump inhibitors, antibiotics, probiotics, or fiber supplementation at the time of sample collection. The study was registered at clinicaltrials.gov (ID: NCT06532110) and approved by the Hamilton Integrated Research Ethics Board (HiREB # 15311). For details see Supplementary Table 1.

### Human sample collection

Fecal samples were collected and stored in anaerobic packages. After transport to the hospital, the samples were aliquoted in an anaerobic chamber^42^. Duodenal aspirates were collected during endoscopy, mixed with 10% glycerol and aliquoted in aerobic conditions^43^. All samples were frozen at −80 °C until analysis.

### Mouse model

Breeding pairs of non-obese diabetic AB° DQ8 (NOD/DQ8) mice^44^ were originally received from Dr. Joseph Murray (Mayo Clinic, USA)^45^. Female and male specific-pathogen free (SPF) mice were bred and housed in the McMaster Central Animal Facility (CAF) and were 8–10-weeks-old and sex-matched for all experimental groups. SPF mice were housed in clean rooms, with *ad libitum* water and an irradiated grain-based diet formulated without gluten (GFD, TD.05620, Envigo; Table 2). Germ-free (GF) NOD/DQ8 mice were generated by two stage embryo transfer and housed within the McMaster Axenic Gnotobiotic Unit (AGU) in flexible film isolators with free access to autoclaved food and water^35^. Diets were autoclaved to ensure sterility. Mice were sacrificed under anaesthesia (Isofluorane) using cervical dislocation before necropsy and tissue collection. All experiments were conducted with approval from the McMaster University Animal Care Committee and McMaster Animal Research Ethics Board (AREB) under the Animal Utilization Protocol (AUP# 210930).

### Gluten sensitization and dietary interventions

Mice were sensitized with gluten using a previously established protocol^21^. Briefly, mice were gavaged with 25μg cholera toxin and 1mg of pepsin/trypsin digested gliadin once a week for 3 weeks. Mice were then switched from a gluten-free diet (TD.05620) to a calorically balanced gluten-containing wheat-based diet (TD.200056) for 3 weeks, and finally returned to GFD for recovery over 2, 6, 10, or 24 weeks. Age-matched naïve controls (non-sensitized, on GFD with no gluten exposure) were used to determine normal and baseline values and compare against experimental groups. Mice receiving fiber-supplemented diets were transitioned over the first week with a 50:50 mixture of no added fiber GFD and either 52.8 g/kg inulin (IN, TD.2100884) diet or 136 g/kg HylonVII (RS, TD.210885) to allow the mice to acclimatize. The total fiber content in the supplemented diets was increased to approximately 15% from a grain-based chow formula with approximately 7.5% dietary fiber. Diet composition is listed in Table 2.

### Evaluation of gluten-immunopathology

Cross sections from the proximal small intestine were fixed in 10% formalin and embedded in paraffin for blinded evaluation using brightfield microscopy (Olympus), as previously described using open-source QuPath software^46,47^. The CD3^+^ intraepithelial lymphocytes (IELs) were quantified by immunohistochemical staining^21^. The primary antibody, polyclonal rabbit antihuman CD3 (S200389–2; Agilent) was diluted 1μL in 1mL of 1% BSA in PBS and incubated overnight. CD3^+^ IELs were visualized with 3,3’-diamonobenzideine (DAB+ substrate chromogen system, K346811–2; Agilent) and counterstained with Mayer’s hematoxylin (S330930–2; Agilent) for 60 seconds. CD3^+^ IELs were quantified in 5 randomly chosen villi tips and reported as the number of IELs per 100 enterocytes. The same paraffin embedded sections were stained with hematoxylin and eosin for villus-height and crypt-depth measurements for the villus-to-crypt ratio (V/C). Ten villi and adjacent crypt pairs were randomly selected and measured. An equally weighted composite VCIEL scoring system was used to combine histological measures and create a standardized baseline around the naïve control group^48^.

At necropsy, the small intestine was washed out and contents collected in 0.05M EDTA in PBS with soy trypsin inhibitor (0.1mg/mL; T9128–1G; Sigma) and phenylmethanesulfonyl fluoride (0.8mM; P7626; Sigma-Aldrich)) to prevent protein degradation. Direct ELISA was performed as previously reported^21,46^. 96-well plates were coated with either transglutaminase in PBS (0.1 μg per well; T5398–2UN; Sigma-Aldrich) or gliadin dissolved in 70% ethanol and diluted in PBS (5 μg per well; G3375; Sigma-Aldrich). Non-specific binding was blocked with 2% BSA in PBS-Tween before 2-hour incubation of 50 μL small intestinal wash solution in triplicate. Detection with 1 hour incubation with HRP-conjugated secondary anti-mouse IgA antibody (1:5000; 1040–05; Southern Biotech) followed TMB substrate (Agilent) reaction for 20 minutes before measuring absorbance at 450nm using a Spectramax M3 plate reader (Molecular Devices). OD values were normalized to the total amount of protein in the sample using a protein quantification assay (Bio-Rad) according to manufacturer’s instructions.

### *Prevotella* cocktail colonization

Ten isolated strains from the *Prevotellaceae* family were obtained from the collection of Dr. Michael Surette’s Lab (McMaster University), based on their reduced abundance in CeD (Table 3). Isolates identities were confirmed with MALDI-TOF and cultured anaerobically in BHI3 for 48 hours with agitation. NOD/DQ8 mice were gavaged with PBS (negative control) or approximately 1 million cells of each strain combined, given on day 1 and 3 of the inulin diet.

### Metabolite determinations

A targeted selected ion monitoring (SIM) gas-chromatography mass-spectrometry (GCMS) protocol was used to quantify SCFA in the small intestinal washes and feces collected at endpoint. GCMS measurements were carried out on an Agilent 5975C inert mass selective detector (MSD) equipped with an Agilent 7890A GC and an Agilent 7693 autosampler (Agilent, Santa Clara, CA, USA), in the Centre for Microbial Chemical Biology (CMCB) at McMaster University. Separation of the analytes was achieved on an Agilent (Santa Clara, CA, USA) DB-1MS UI (30 m × 0.25 mm × 0.25μm) column. The injector, ion source, quadrupole and the GCMS interface temperatures were 260, 230, 150, and 280 °C, respectively. The flow rate of helium carrier gas was kept at 1 mL/min. 1 μL of derivatized sample was injected with a 4.5-minute solvent delay time and split ratio of 10:1. The initial column temperature was 40 °C and held for 5 minutes, ramped to 165 °C at the rate of 10 °C/min, and then increased to 310 °C at the rate of 100 °C/min and kept at this temperature for 3 minutes. The total run time was 22 minutes. The ionization was carried out in the electron impact (EI) mode at 70 eV. The MS data were acquired in the SIM mode. The identification of compounds was confirmed by injection of pure standards and comparison of the retention time and target ions.

### RNA extraction and quantitative reverse-transcription PCR (RT-qPCR)

Samples from the duodenum, jejunum, ileum, and proximal colon were collected in RNALater (Invitrogen) before RNA extraction using RNEasy Mini kit (Qiagen) according to manufacturer’s instructions. Extracted RNA was used for cDNA synthesis using iScript Reverse Transcriptase (Bio-Rad) with SsoFast EvaGreen Supermix (BioRad) according to manufacturer’s instructions. The qPCR cycling consisted of initial denaturation at 95 °C for 3 minutes, followed by 40 cycles of denaturation at 95 °C for 10 seconds and annealing/extension at 65 °C for 30 seconds. GAPDH was used as an endogenous control for normalization of gene expression, and data was reported as the fold change in expression in response to inulin compared to GFD alone. Validated primer sequences for mouse GPR41 (142345193c1) and GPR41 (22122727a1) were obtained from Primer Bank^49^.

### Microbiota sequencing and predicted microbial function.

After genomic DNA extraction, an established protocol consisting of a 2-stage nested polymerase chain reaction (PCR) to amplify the variable regions 3–4 of the 16S rRNA gene from low biomass samples was used to sequence the mouse duodenum and human aspirates using the MiSeq Illumina platform^9^. Sequences were then processed in R, version 4.4.2 using the package Divisive Amplicon Denoising Algorithm 2 (DADA2^50^) and the SILVA reference database, version 138.1^51^. FastTree 2^52^ was used to calculate a phylogenetic tree of sequences and data were explored using the phyloseq package^53^. Functional predictions based on 16S rRNA sequences were made using PICRUSt2^54^, annotated using the Kyoto Encyclopedia of Genes and Genomes database^55^. Beta-Diversity was calculated using the Aitchison distance^56^ and differences between groups were calculated using PERMANOVA. Taxonomic and functional microbial differences were evaluated using a generalized linear mixed mode with a negative binomial distribution^57^ with adjustment for age and sex as confounding variables for patient data. Statistical differences between groups were identified using estimated marginal means using the emmeans package^58^. 16S rRNA gene sequences were deposited in Sequence Read Archive (PRJNA1256891)

**FoodSeq** (Sequencing, bioinformatic pipeline, and diversity analysis).

We performed DNA extraction on randomized fecal samples using the DNeasy PowerSoil Pro kit (Qiagen) following manufacturer’s instructions. We prepared libraries using a two-step PCR process^59^ using *trnL* g and h primers^60–62^. Primary PCR amplification was conducted as previously described^62^. Secondary PCR amplification contained 5 μL each of 2.5 μM forward and reverse indexing primers^62^, 25 μL of 2X KAPA HiFi HotStart Ready Mix, 0.5 μL of 100X SYBR Green I, 9.5 μL nuclease-free water, and 5 μL of primary PCR product diluted 1:100 in nuclease-free water. Amplicon libraries were then cleaned and pooled as previously described^62^. Paired-end sequencing was carried out on an Illumina MiniSeq using a 300-cycle Mid kit (Illumina).

We demultiplexed, trimmed, filtered, and denoised sequence reads using a QIIME2^63^ pipeline v. 2023.5.1 with DADA2^50^ plugin. Sequence reads were aligned to a custom *trnL* reference database^62^ (available at https://github.com/LAD-LAB/mb-pipeline/blob/main/reference/trnLGH.fasta) and taxonomic assignments were made using assignSpecies() in the DADA2 R package v. 1.30.0.

We calculated observed richness and Shannon diversity using estimate_richness() in the phyloseq^52^ R package v. 1.46.0 for each sample after removing taxa unassigned to any dietary species in our reference database. Principal component analysis was performed using prcomp(), centered and not scaled, on CLR-transformed relative abundance data, prior to unassigned taxa removal. A distance matrix was generated using Euclidean distance calculations. PERMANOVA was performed using adonis2() in the vegan R package v. 2.6.6.1^64^ adjusting for age, sex, ethnicity/race, and posthoc pairwise comparisons using pairwiseAdonis() v. 0.4.1^65^, adjusting p-values using the default Bonferroni method. The *trnL* gene sequences were deposited in Sequence Read Archive (PRJNA1256896)

### Statistical analysis.

Graphpad Prism was used for most analyses. Data are presented for each individual sample (human or mouse). All data were first tested for statistical outliers (ROUT, Q=1%) and then for normality using the Kolmogorov-Smirnov test, such that non-parametric tests were used for non-normally distributed data. For comparisons between two groups, the Student’s *t-*test or the non-parametric Mann-Whitney was used. For three or more groups, one-way analysis of variance (ANOVA) was used with Tukey’s post hoc test, or the nonparametric Kruskal-Wallis followed by Dunn’s test. *p*<0.05 was used to determine statistically significant effects.

## Supplementary Material

1

Supplementary Files

This is a list of supplementary files associated with this preprint. Click to download.

- Wulczynskietal.CeDFiberSupplementaryfigures.pdf

## Figures and Tables

**Table 1:** Patient demographic summary and number of samples collected for analysis.

			Questionnaires and Samples available				
Diagnosis	Mean age	Number / Sex	FFQ	GSRS (n); mean score	Fecal FoodSeq	Fecal SCFA	Duodenal aspirate 16S
**Healthy controls CeD**	37.5	26 (14 F: 12 M)	n = 5	(n = 23); 20	n =23	n = 18	n = 17
	38.8	16 (12 F: 4 M)	n = 6	(n = 9); 45	n = 15	n = 11	n = 7
**Treated CeD**	43	11 (8 F:3 M)	n = 7	(n = 8); 37	n = 11	n = 8	n = 7

Average age, sex distribution, and number of samples available for each group. Individual demographic information is further detailed in Supplementary Table 1.

CeD= celiac disease, FFQ= food frequency questionnaire; GSRS= Gastrointestinal Rating Symptoms Scale; Fecal FoodSeq=fecal plant DNA; Fecal SCFA= fecal short chain fatty acids.

**Table 2:** Composition of mouse diets.

Diets and Nutrient Information		TD.05620	TD.200056	TD.210884	TD.210885
		GFD	Wheat diet	Inulin	HylonVII
	Protein	20.9	20.9	21.9	20.1
% kcal from	Carbohydrate	65.9	65.9	64.3	67.8
	Fat	13.2	13.2	13.8	12.1
Kcal/g		3.2	3.2	3	3.1
Dietary Fiber		5–10%	5–7%	15.80%	15.50%
Ingredients (g / kg)					
Corn		500	N/A	500	364
Soybean Meal		270	265	270	270
Soybean Hull		40	40	40	40
DL-Methionine, FG (99%)		1.5	1.5	1.5	1.5
Maltodextrin		115.73	115.73	62.93	115.73
Lard		25	25	25	25
Mineral Mix, U.S. Rodent (99115)		2.5	2.5	2.5	2.5
Calcium Carbonate, FG (38%)		13	13	13	13
Dicalcium Phosphate, FD, 18.5% P, 21% C)		12	12	12	12
Magnesium Oxide, FD (58%)		2.2	2.2	2.2	2.2
Sodium Chloride, iodized		10	10	10	10
Vitamin Mix, U.S. Rodent (99114)		2.5	2.5	2.5	2.5
Vitamin Mix, U.S. Supplement (99116)		2.5	2.5	2.5	2.5
Choline Chloride, FG (60%)		2.07	2.07	2.07	2.07
Locust Bean Gum w/ gelatin		1	1	1	1
Wheat		N/A	375	N/A	N/A
Corn Starch		N/A	116	N/A	N/A
Cellulose		N/A	1.8	N/A	N/A
Corn Oil		N/A	12.2	N/A	N/A
Inulin		N/A	N/A	52.8	N/A
High Amylose Corn Starch		N/A	N/A	N/A	136

Custom diets were based on our mouse gluten-free diet (GFD) formulated by Envigo (Inotiv) to substitute macromolecule sources (e.g. corn vs wheat) or specific nutrients (e.g. inulin or HylonVII)

**Table 3:** *Prevotella* isolates and sources

			# Gene copies	
Bacteria	ID#	Source	α-Amylase	Fructan β-fructosidase
*Alloprevotella alloprevotella*	GC2143	Feces	1	1
*Prevotella copri*	GC2072	Feces	1	2
*Prevotella denticola*	GC1774	Nasal	1	1
*Prevotella histicola*	GC335	Nasal	1	2
*Prevotella intermedia*	GC330	Sputum	1	1
*Prevotella melanogenica*	GC149	Sputum	1	2
*Prevotella nanceiensis*	GC155	Sputum	1	1
*Prevotella nigrescens*	GC308	Saliva	1	1
*Prevotella oralis*	GC309	Sputum	1	1
*Prevotella salivae*	GC332	Saliva	1	1

All bacterial strains were obtained from the genome collection (GC) of Dr. Michael Surette (McMaster University). Bacteria were isolated from healthy humans.
