# Supplementary material for: Small intestinal microbial fiber metabolism dysfunction in celiac disease
Source: Res Sq. 2025 May 12:rs.3.rs-6572358. Preprint. [Version 1] doi: 10.21203/rs.3.rs-6572358/v1 (PMC12136232; doi:10.21203/rs.3.rs-6572358/v1)
Supplement: 1 [file NIHPPRS6572358V1-supplement-1.pdf]

Supplementary Figure 1: Individual study participant duodenal microbiota composition.

a Abundance plot of duodenal genera in each individual participant, with legend. Healthy controls (n= 17), CeD (n= 7), and T-CeD (n= 7). **b** Relative abundance of *Prevotella sp.* predicted to have the largest metagenomic contribution to microbial carbolytic enzymes. Multiple groups were compared using one-way ANOVA followed by Tukey's test.

Supplementary Figure 2: Individual heterogeneity of dietary plant DNA in feces.

a Abundance plot of fecal plant taxa in each individual participant, with legend. **b** Estimated daily fiber intake and self-reported gastrointestinal symptoms ratings scores were not correlated. Healthy controls (n= 5), CeD (n= 6), and T-CeD (n= 7) were pooled together for analysis (n= 18).

Supplementary Figure 3: Gluten-immunopathology resolves by week 10 of GFD.

a Protocol for gluten-immunization, challenge, and GFD-recovery. 8–10-week-old specific-pathogen free (SPF) NOD/DQ8 mice were immunized to gluten by weekly oral gavage with 25 µg cholera toxin and 1 mg of pepsin/trypsin digested gliadin. Mice were then given gluten-containing wheat diet for 3 weeks before being returned to GFD for recovery. Mice (n = 4-8 per group) were sacrificed after 0, 2, 6, 10, and 24 weeks of recovery and compared to naïve controls (non-sensitized and without gluten exposure). **b** $CD3^+$ intraepithelial lymphocytes numbers over time during GFD recovery. **c** Villus-to-crypt ratio over time during GFD recovery. **d**, Composite VCIEL scores over time during GFD recovery. Dashed line represents the baseline determined from naïve controls. **e** Representative histological images of jejunal tissue over time during GFD recovery. For all data, multiple groups were compared using one-way ANOVA followed by Tukey's test, significant differences (p<0.05) between groups were indicated with a compact

letter display. (where A indicates statistical difference from B, and AB indicates a group is not different from A or B, etc).

Supplementary Figure 4: Small intestinal antibodies against gliadin or TG2 s in gluten-sensitized NOD/DQ8 mice over extended recovery.

a Small intestinal IgA antibody concentration over time during GFD recovery. Dashed line represents the positivity threshold determined as 3 standard deviations above the mean naïve controls. **b** Spearman correlation between small intestinal anti-TG2 and anti-gliadin IgA in mice fed GFD over time for up to 24 weeks. **c** Small intestinal IgA antibody concentrations over time during GFD recovery. Dashed line represents the positivity threshold determined as 3 standard deviations above the mean naïve controls. **d** Spearman correlation between small intestinal anti-TG2 and anti-gliadin IgA in mice fed no added fiber GFD, inulin, or HylonVII supplemented over six (triangles), 10 (circles), or 12 (squares) weeks of recovery. For **a, c,** data was compared using multiple groups were compared using one-way ANOVA followed by Tukey's test.

Supplementary Figure 5: Resolution of gluten-immunopathology by week 10 of GFD in NOD/DQ8 mice.

a Faith's Phylogenetic Diversity α-diversity of duodenal microbiota after six weeks of recovery on either no added fiber GFD (grey), inulin (blue), or HylonVII (yellow). Multiple groups were compared using one-way ANOVA followed by Tukey's test. **b** Relative abundances of bacterial genera in the mouse duodenum after 6 weeks of recovery, with legend.

Supplementary Figure 6: Effect of microbiota and fiber on fecal SCFA and expression of intestinal SCFA receptors

a Fecal SCFA after 3 weeks of no added fiber GFD (white) or inulin diet (blue) in GF (squares) and SPF (circles) mice. (n= 5 / group) multiple groups were compared using one-way ANOVA followed by Tukey's test. **b** Relative change in SCFA receptor *Gpr*43 measured in tissue samples from the duodenum, jejunum, ileum, and colon of GF and SPF mice when supplemented inulin. Data is represented as the fold-change in *Gpr*43 expression when fed inulin compared to no added fiber diets as control for GF and SPF mice. **c** Expression of *Gpr*43 in the ileum. For each location, a Student's T Test compared *Gpr*43 expression in mice fed GFD with no added fiber or with inulin; for GF or SPF mice.

Supplementary Table 1: Individual patient demographic information

Diagnosis	Sex	Age	HLA	Marsh Score	Elevated TG2[1]
Control	M	60	ND	ND[2]	No
Control	F	58	ND	ND	No
Control	F	63	ND	ND	No
Control	M	63	ND	ND	No
Control	M	63	ND	ND	No
Control	F	65	ND	0	No
Control	M	42	ND	0	No
Control	F	36	ND	0	No
Control	F	24	ND	0	No
Control	M	25	ND	0	No
Control	F	25	ND	0	No
Control	F	23	ND	0	No
Control	F	26	ND	0	No
Control	M	27	ND	0	No
Control	M	57	ND	0	No
Control	F	37	ND	0	No
Control	F	28	ND	0	No
Control	F	25	ND	0	No
Control	M	28	ND	0	No
Control	M	29	ND	0	No
Control	M	25	ND	0	No
Control	F	19	ND	0	No
Control	F	36	ND	0	No
Control	M	33	ND	0	No
Control	F	31	ND	0	No
Control	M	28	ND	0	No
New CeD	M	33	ND	3c	Yes*
New CeD	F	26	ND	3a	Yes
New CeD	M	46	DR3-DQ2 homozygotic	3a	Yes
New CeD	F	34	ND	3a	Yes
New CeD	M	55	DR3-DQ2 heterozygotic	3c	Yes
New CeD	F	30	DR3-DQ2 heterozygotic	3a	Yes
New CeD	M	41	DR3-DQ2 heterozygotic	1	Yes
New CeD	F	62	DR3-DQ2 heterozygotic	3a	Yes
New CeD	F	22	DR3/DR7-DQ2 homozygotic	3a	Yes*
New CeD	F	35	ND	3b	Yes*
New CeD	F	38	DQ2 present	3a	Yes
New CeD	F	68	DQ2 present	3a	Yes

New CeD	F	46	DQ2 present	3a	Yes
New CeD	F	19	DQA5/DQ2 present	3a	Yes
New CeD	F	26	ND	3a	Yes
New CeD	F	40	DQ2 present	3b	Yes
Treated CeD	M	52	DR3-DQ2 heterozygotic	3a	No
Treated CeD	F	35	DR3-DQ2 heterozygotic	3a	No
Treated CeD	F	31	ND	3a	No
Treated CeD	F	27	DR3-DQ2 heterozygotic	1	No
Treated CeD	M	52	DR3-DQ2 heterozygotic	1	No
Treated CeD	M	54	DR3-DQ2 homozygotic	0	No
Treated CeD	F	29	DR3-DQ2 heterozygotic	0	No
Treated CeD	F	27	DR7-DQ2/DQ7 heterozygotic	3a	No
Treated CeD	F	58	ND	3a	No
Treated CeD	F	46	ND	0	No
Treated CeD	F	62	DQ2 present	0	No

The age, sex, and diagnostic criteria for each individual participant.

[1] TG2 was elevated > 5 times above normal in the majority of CeD patients. . Three CeD patients indicated with * were only 2 times above the positive threshold. All healthy controls and T-CeD patients were below the positive threshold, despite 5 T-CeD presenting persistent villous atrophy.

[2]ND – Not determined, due to lack of participant consent for genetic analysis or sample not collected.
